# Low Plasma Ergothioneine Predicts Cognitive and Functional Decline in an Elderly Cohort Attending Memory Clinics

**DOI:** 10.3390/antiox11091717

**Published:** 2022-08-30

**Authors:** Liu-Yun Wu, Cheuk Ni Kan, Irwin K. Cheah, Joyce Ruifen Chong, Xin Xu, Henri Vrooman, Saima Hilal, Narayanaswamy Venketasubramanian, Christopher P. Chen, Barry Halliwell, Mitchell K.P. Lai

**Affiliations:** 1 Department of Pharmacology, Yong Loo Lin School of Medicine, National University of Singapore, Singapore 117599, Singapore; 2 Memory Aging and Cognition Centre, National University Health System, Singapore 117577, Singapore; 3 Department of Biochemistry, Yong Loo Lin School of Medicine, National University of Singapore, Singapore 117596, Singapore; 4 Life Science Institute, Neurobiology Programme, Centre for Life Sciences, National University of Singapore, Singapore 117456, Singapore; 5 School of Public Health and the Second Affiliated Hospital, Zhejiang University School of Medicine, Hangzhou 310058, China; 6 Departments of Epidemiology and Radiology and Nuclear Medicine, Erasmus University Medical Center, 3025 Rotterdam, The Netherlands; 7 Saw Swee Hock School of Public Health, National University of Singapore, Singapore 117549, Singapore; 8 Raffles Neuroscience Centre, Raffles Hospital, Singapore 188770, Singapore; 9 Department of Psychological Medicine, Yong Loo Lin School of Medicine, National University of Singapore, Singapore 119228, Singapore

**Keywords:** ergothioneine, biomarker, cognitive decline, dementia, cerebrovascular disease, neurodegeneration

## Abstract

Low blood concentrations of the diet-derived compound ergothioneine (ET) have been associated with cognitive impairment and cerebrovascular disease (CeVD) in cross-sectional studies, but it is unclear whether ET levels can predict subsequent cognitive and functional decline. Here, we examined the temporal relationships between plasma ET status and cognition in a cohort of 470 elderly subjects attending memory clinics in Singapore. All participants underwent baseline plasma ET measurements as well as neuroimaging for CeVD and brain atrophy. Neuropsychological tests of cognition and function were assessed at baseline and follow-up visits for up to five years. Lower plasma ET levels were associated with poorer baseline cognitive performance and faster rates of decline in function as well as in multiple cognitive domains including memory, executive function, attention, visuomotor speed, and language. In subgroup analyses, the longitudinal associations were found only in non-demented individuals. Mediation analyses showed that the effects of ET on cognition seemed to be largely explainable by severity of concomitant CeVD, specifically white matter hyperintensities, and brain atrophy. Our findings support further assessment of plasma ET as a prognostic biomarker for accelerated cognitive and functional decline in pre-dementia and suggest possible therapeutic and preventative measures.

## 1. Introduction

With rapidly aging global populations, cognitive decline and dementia have become leading causes of functional disability among the elderly. An estimated 55 million people worldwide currently suffer from dementia, with the figure expected to triple by 2050 [1]. Moreover, nearly four times as many people are affected by mild cognitive impairment (MCI), which puts them at a greater risk of subsequently developing dementia [2]. In the coming decades, this is projected to significantly increase the healthcare burden and morbidity for patients, caregivers, and societies [3]. As an essential aspect of healthy aging, maintaining cognitive function has therefore received increasing public health attention. A number of nutritional and lifestyle factors have been identified as potentially modifiable risk factors for cognitive decline [4]. Research efforts in modifiable pathophysiological targets have focused on oxidative stress, which can lead to the onset and progression of cognitive impairment and dementia [5,6], but which can also be ameliorated to some degree by increased physical activity [7] and good nutrition [8]. One of the major causes of increased oxidative stress observed in age-related cognitive impairment may be a decrease in certain antioxidant defence mechanisms [9]. In particular, decreased plasma levels of several antioxidant micronutrients have been associated with dementia [10,11]. In line with these observations, a number of clinical studies examining the use of antioxidants to treat cognitive impairment have been attempted. Unfortunately, trials on supplementation with conventional antioxidants (e.g., vitamins C and E) have shown little efficacy in preventing or delaying cognitive decline [12]. This suggests that there could be other factors affecting the antioxidant response of these supplements, such as rates of metabolism and excretion, bioavailability within the central nervous system (CNS), whether they actually function as antioxidants under physiological conditions [13], and so on, which highlight possible reasons for the failure of earlier antioxidant trials, as well as a need for the identification of new therapeutic targets for developing more effective treatment strategies.

One promising molecular target is ergothioneine (ET), a naturally occurring dietary thiol/thione with antioxidant properties that is present in a wide variety of foods, especially in mushrooms [14,15]. In humans, ET is rapidly taken up and accumulated in specific cells and tissues (including the brain) that express OCTN1 (organic cation transporter novel, type 1), a selective high-affinity transporter for ET [16,17,18]. At physiological pH, ET exists predominantly in the thione form, which confers stability and resistance to autoxidation [19]. However, numerous in vitro studies have demonstrated the antioxidant activity of ET and ability to neutralize reactive oxygen/reactive nitrogen species (ROS/RNS) [15,20,21]. Although the exact physiological function of ET has yet to be elucidated, there is growing evidence supporting its potential neuroprotective and cognition-enhancing capabilities [14,21]. Studies in cells and animal models have shown that ET can protect against ROS formation and neuronal injury following insults by various neurotoxic agents such as cisplatin and amyloid beta peptides [22,23,24]. Oral administration of ET has been shown to improve cognition in beta amyloid- or D-galactose-induced mice models of cognitive impairment [25,26]. Furthermore, clinical studies have reported significantly lower plasma ET in patients with MCI, Alzheimer’s dementia (AD), and vascular dementia (VaD), as compared with cognitively normal controls [11,27,28]. Additionally, our group has recently established that decreased plasma ET was associated with neuroimaging measurements of cerebrovascular disease (CeVD) as well as neurodegeneration [11].

Given its established antioxidant and cytoprotective properties, ET deficiency has thus been suggested as a potential modifiable risk factor linked to cognitive decline [27,29,30]. However, the cross-sectional design of the previous studies [11,27,28] is limited to investigations of baseline clinical diagnoses, and these studies did not address whether low ET (1) correlated with baseline cognition or (2) predicted disease progression. Indeed, there is currently a paucity of data on the longitudinal associations between ET and cognitive trajectory, with only one metabolomics-based study identifying a link between peripheral ET (together with several other metabolites) and subsequent cognitive decline [31]. Until now, it has also been unclear whether ET might be associated with perturbations of specific cognitive domains, including executive function, language, visuospatial function, and memory, among others. Furthermore, the pathophysiological mechanisms mediating the putative associations between plasma ET and cognitive decline remain unexplored. Therefore, the present study of a memory-clinic cohort with up to five years follow-up aimed to determine (1) how baseline plasma ET may be associated with cross-sectional cognitive performance as well as longitudinal cognitive and functional changes and (2) the extent to which associations between plasma ET alterations and cognitive decline are mediated by CeVD and neurodegeneration.

## 2. Materials and Methods

### 2.1. Study Population

Data used in the present study were obtained from an ongoing prospectively assessed cohort, consisting of patients recruited from two study sites in Singapore (National University Hospital and St. Luke’s Hospital). Inclusion and exclusion criteria have been described elsewhere [32]. A total of 513 participants were recruited between August 2010 to July 2019 with blood plasma samples collected at their first study visit, of whom 470 were included in this study based on available clinical and neuropsychological data both at baseline as well as annual follow-up visits (see Supplementary Figure S1) with a maximum follow-up period of five years (average follow-up = 3.7 [SD ± 1.5] years). Institutional Review Board approval was received from the National Healthcare Group (DSRB reference 2010/00017; study protocol DEM4233) and was conducted in compliance with the guidelines in the Declaration of Helsinki. All participants provided written informed consent before recruitment.

All participants underwent detailed neuropsychological testing, neuroimaging, and clinical evaluations for classification into three diagnostic categories at baseline, namely, (1) No cognitive impairment (NCI): no objective cognitive impairment based on formal neuropsychological tests; (2) Cognitive impairment, no dementia (CIND): objective impairment in at least one cognitive domain on a neuropsychological test battery [33] while remaining functionally independent and not meeting Diagnostic and Statistical Manual of Mental Disorders Fourth Edition (DSM-IV) criteria for dementia; or (3) Dementia clinically diagnosed according to DSM-IV criteria. Further etiological diagnoses of dementia were based on the National Institute of Neurological and Communicative Disorders and Stroke and the Alzheimer’s Disease and Related Disorders Association (NINCDS-ADRDA) criteria for Alzheimer’s disease (AD) [34] and the National Institute of Neurological Disorders and Stroke and Association Internationale pour la Recherche’et l’ Enseignement en Neurosciences (NINDS-AIREN) criteria for vascular dementia (VaD) [35]. Annual clinical assessments included a comprehensive neuropsychological battery (see below) as well as the Clinical Dementia Rating Sum-of-Boxes (CDR-SOB) scale [36] used to determine functional decline.

### 2.2. Plasma Ergothioneine Measurements

Blood was drawn from study participants into ethylenediaminetetraacetic acid (EDTA) tubes and processed by centrifugation at 2000× *g* for 10 min at 4 °C, followed by extraction of the upper plasma layer and storage at −80 °C until use. The plasma ergothioneine concentration was measured using a high sensitivity and specificity liquid chromatography tandem-mass spectrometry (LC–MS/MS) method as previously described [11,37]. The analytical procedures were performed by investigators blinded to all demographic, clinical, and neuroimaging data.

### 2.3. Neuropsychological Assessments

All patients completed brief cognitive testing with the Mini-Mental State Examination (MMSE) as well as a detailed neuropsychological battery based on the recommendation of the National Institute of Neurological Disorders and Stroke and the Canadian Stroke Network [38] at baseline and during each follow-up visit. The component tests for assessing the six cognitive domains (executive function, language, visuomotor speed, visuospatial function, attention, and memory) are detailed in Supplementary Table S1. Individual raw test scores were converted to standardized Z-scores based on the means and SDs of the control group (NCI). The score for each cognitive domain was computed by averaging the Z-scores of individual tests and standardized using the composite mean and SD of the control group. To obtain the global cognition score for each patient, the domain-based Z-scores were averaged and standardized using the mean and SD of the control group. At follow-up, global and domain-based cognitive Z-scores were calculated using the means and SDs of the control group at baseline.

### 2.4. Covariate Assessments

A detailed questionnaire was administered to all participants to collect information on age, sex, race, and highest attained education. Physical examination included height, weight, and blood pressure measurements. Risk factors, such as hypertension, hyperlipidemia, diabetes mellitus, and cardiovascular diseases, were ascertained from clinical interviews and medical record searches and classified as present or absent. Hypertension was defined as systolic blood pressure ≥140 mm Hg and/or diastolic blood pressure ≥90 mm Hg, or history of antihypertensive medication use. Diabetes mellitus was defined as glycated haemoglobin (HbA1c) of 6.5% or higher, or on medication. Hyperlipidemia was defined as total cholesterol levels of 4.14 mM or more, or on medication. Cardiovascular disease was classified as a previous history of atrial fibrillation, congestive heart failure, or myocardial infarction. Apolipoprotein E (APOE) genotyping was performed as described previously [39], and APOE ε4 carrier status was defined as having at least one ε4 allele. Body mass index (BMI) was calculated as mass (kg)/height squared (m^2^).

### 2.5. Neuroimaging

Magnetic resonance imaging (MRI) scans were performed at baseline on a 3-Tesla Siemens Magnetom Trio Tim scanner, using a 32-channel head coil, at the Clinical Imaging Research Centre, National University of Singapore. The standardized neuroimaging protocol includes T1-weighted Magnetization Prepared Rapid Gradient Recalled Echo, Fluid Attenuated Inversion Recovery (FLAIR), T2-weighted, and Susceptibility Weighted Imaging sequences. MRI markers of CeVD were defined based on the Standards for Reporting Vascular Changes on Neuroimaging (STRIVE) criteria [40]: Cortical infarcts were defined as hypodense lesions interrupting the cortex grey/white junction; lacunes were defined by the presence of hypodense focal lesions measuring ≥3 mm and <15 mm; WMH were defined as hyperintense on T2 and FLAIR sequences and hypointense on T1 weighted images and were graded using the Age-Related White Matter Changes (ARWMC) scale [41]. Quantitative MRI analyses of the total intracranial volume, global cortical thickness, and hippocampus volume were quantified by automatic segmentation at the Department of Medical Informatics, Erasmus University Medical Center, The Netherlands (FreeSurfer, v.5.1.0) on T1 weighted images (TR = 7.2 ms, TE = 3.3 ms, matrix = 256 × 256 × 180 mm^3^). Image preprocessing and the tissue classification algorithm have been described elsewhere [42].

### 2.6. Statistical Analyses

Statistical analyses were performed using SPSS Statistics (version 26, IBM Inc., Armonk, NY, USA) and R (version 4.0.2). Baseline differences between patients with dementia and those without were compared using Student’s t-test, the Mann–Whitney U test, or the Chi-square test as appropriate. Linear and linear mixed-effects (LME) regression models were used to assess associations of baseline plasma ET with cross-sectional and longitudinal changes in cognitive function, respectively. We evaluated models with baseline plasma ET concentrations treated both continuously (log-transformed due to skewed distribution) and dichotomously (stratified based on median cut-off = 784 nM). Regression models were adjusted for age, gender, education, APOEε4 allele status, hypertension, diabetes, cardiovascular disease, as well as (in longitudinal analysis) baseline MMSE scores. All LME models included random effects for the patient and fixed effects for baseline plasma ET, time, the interaction between baseline ET and time, and the adjustment covariates. The models were fitted using maximum likelihood estimation with a random intercept and slope included. These analyses were performed for cognitive z-scores and CDR-SOB scores as outcomes in all patients and separately for patients with and without dementia. In addition, logistic regression models were constructed to determine the association between baseline plasma ET levels and functional decline (defined as ≥1-point increase in CDR-SOB scores during follow-up).

Finally, as mediation models allow for quantitation of the extent to which an intermediary variable participates in the observed associations between the independent and dependent variables, we performed mediation analyses to investigate the mediation effect of imaging CeVD and neurodegeneration markers on the association between baseline plasma ET and global cognition decline, in which bootstrapping methods were used to estimate the 95% CI. In each model, plasma ET was included as the independent variable, cognitive change (i.e., estimated rates of change or slope from LME models) as the dependent variable, and MRI markers as mediator variables, adjusting for the total intracranial volume and the other covariates as in the previous analyses. All tests were 2-sided with a significance level of *p* < 0.05.

## 3. Results

### 3.1. Baseline Demographic and Disease Factors of the Study Cohort

Table 1 shows the baseline demographic characteristics of the 470 participants included in this study. Among them, 281 (59.8% of total) were categorized as non-demented (including both NCI [n = 88, age range = 52–85] and CIND [n = 193, age range = 55–88]) and 189 (40.2%) as demented (including AD [n = 146] and VaD [n = 43], age range = 55–91). Compared to the non-demented group, patients with dementia were older, less educated, had a higher frequency of APOE ε4 genotype, hypertension, and higher burden of cerebrovascular disease (i.e., WMH, lacunes, and cortical infarcts), as well as more pronounced brain atrophy (denoted by reduced cortical thickness and hippocampus volume). Baseline plasma ET concentrations were significantly lower in dementia patients compared with those without dementia. For all participants, plasma ET levels were lower in male participants and APOE ε4 carriers (*p* < 0.001 and *p* = 0.004, respectively, Mann–Whitney U tests) and were negatively associated with age (Spearman’s *r* = −0.18, *p* < 0.001), hypertension, diabetes, and cardiovascular disease (*p* = 0.006, *p* = 0.030 and *p* = 0.001, respectively, Mann–Whitney U tests), but positively associated with the education level (Spearman’s r = 0.20, *p* < 0.001), but not with BMI or hyperlipidemia (both *p* > 0.1). Therefore, age, gender, education, APOE ε4 status, hypertension, diabetes mellitus, and cardiovascular disease were included as covariates in subsequent analyses.

### 3.2. Associations of Plasma ET Levels with Cognition at Baseline

**Figure 1:**
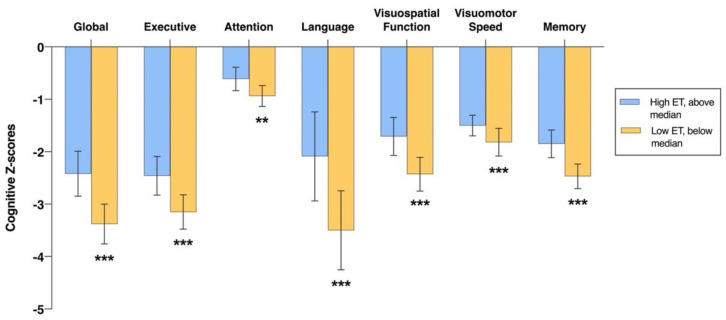
Baseline measures of global and domain-specific cognitive z-scores for high and low ET subjects. Bar graphs represent the estimated marginal means from the linear regression models, with error bars representing 95% confidence intervals. Group median ET was used as a cut-off point for High ET vs. Low ET designations. Analyses are shown with adjustments for age, gender, education, APOE ε4 status, hypertension, diabetes, and cardiovascular disease. ** and *** indicate significant associations based on multiple linear regression at *p* < 0.01 and *p* < 0.001, respectively. Abbreviations: ET = ergothioneine.

In cross-sectional analyses, individuals with low ET levels (below median concentration) showed significantly worse performance in global cognitive function and all six individual cognitive domains at baseline, when compared to the higher ET group (above median concentration), after adjustments for demographic and vascular risk factors (Figure 1). Similar results were obtained when log-transformed continuous ET values were linearly regressed with z-scores of the cognitive tests (Table 2). Within the non-demented subgroup, associations were observed between baseline ET concentrations and cognition in global function as well as four cognitive domains (executive, visuospatial, visuomotor speed, and memory; Supplementary Table S2), while only the association with memory remained significant when ET was treated as a dichotomous variable (Supplementary Figure S2). In contrast, within the dementia subgroup, ET was associated with the visuospatial function domain when tested as either a continuous or a dichotomous variable (Supplementary Figure S2 and Table S2).

### 3.3. Associations of Baseline Plasma ET Levels with Cognitive Trajectories and Functional Decline

**Figure 2:**
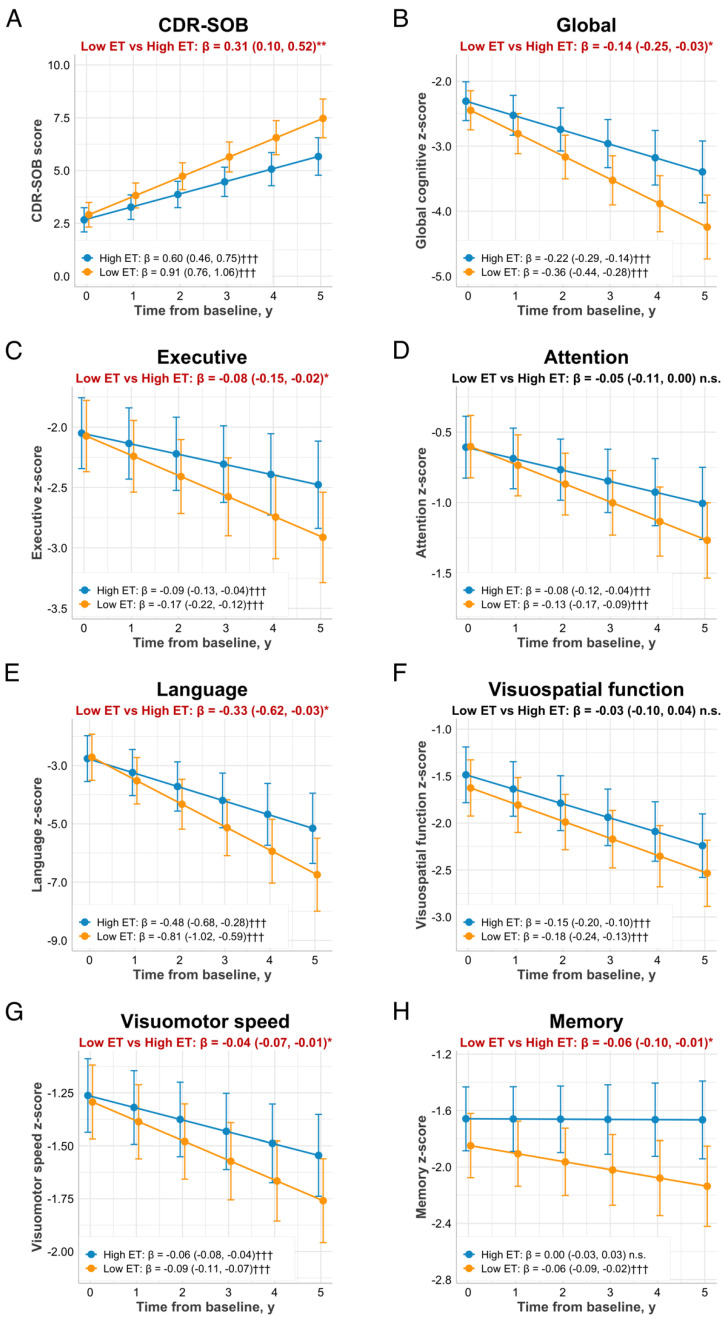
Trajectories of cognitive and functional decline in relation to baseline plasma ET status. Estimated means of CDR-SOB scores (**A**) and global/domain-specific cognitive z-scores (**B**–**H**) with 95% CI are presented for those with high baseline ET (above group median; blue lines) and low baseline ET (below group median; orange lines). β-coefficients (95% CI) were derived from linear mixed-effect models adjusted for age, gender, education, APOE ε4 status, hypertension, diabetes, cardiovascular disease, and baseline MMSE. * and ** (in red text) indicate significantly different rates of change in cognitive scores between low ET vs. high ET patients at *p* < 0.05 and *p* < 0.01, respectively. ††† indicates a significant increase in CDR-SOB or a significant decline in cognitive z-scores over time (slope) at *p* < 0.001. Abbreviations: CDR-SOB = Clinical Dementia Rating Sum-of-Boxes, CI = confidence interval, ET = ergothioneine; MMSE = Mini-Mental State Examination, n.s. = not significant.

We then investigated whether low baseline plasma ET levels predicted subsequent cognitive and functional decline (mean follow-up = 3.7 years). The effects of baseline plasma ET on trajectories for CDR-SOB scores and neuropsychological z-scores are shown in Figure 2 with adjustment for baseline cognition. Rates of change in scores (i.e., slope coefficients) were compared for low versus high ET groups. Patients with low baseline ET levels had a faster progression rate in CDR-SOB scores over five years (β: 0.31, 95% CI: 0.10, 0.52; *p* = 0.004) as compared to those in the high ET group (Figure 2A). Subgroup analyses revealed a non-significant effect of low ET on CDR-SOB increase in the non-demented subgroup (β: 0.19; 95% CI: −0.03, 0.41; *p* = 0.094), while no trend was observed in patients with dementia (*p* = 0.877, Supplementary Figure S3A). Notably, logistic regression analyses revealed that in the non-demented subgroup, individuals with lower ET levels were more likely to have ≥1-point increase in CDR-SOB scores over the observed follow-up duration (odds ratio: 0.21, 95% CI: 0.06, 0.76, *p* = 0.018). However, these associations were not found in the dementia subgroup (*p* = 0.413, Supplementary Figure S4).

Global cognitive function decreased in both low and high ET groups over time, with significantly higher rates among patients with low baseline ET levels (β: −0.14, 95% CI: −0.25, −0.03; *p* = 0.012 vs. the high ET group, Figure 2B). There were trends towards faster rates of decline in the low ET group in all individual cognitive domains (β ranges from −0.33 to −0.04; all *p* < 0.05 vs. the high ET group) except for attention (which reached borderline significance of *p* = 0.057) and visuospatial function (Figure 2C–H). When ET levels were analyzed as a continuous variable, similar associations between baseline ET levels and longitudinal cognitive decline across multiple domains were observed (Table 3). When stratified by dementia status, the association of low baseline ET and declining cognitive function was only observed among non-demented patients, specifically in the visuomotor speed domain (*p* = 0.018, Supplementary Figure S3G). Although scores in global cognition decreased more steeply in non-demented patients with low ET (with a mean range of −0.21 to −0.07) compared to those with high ET (with a mean range of −0.11 to 0.00), the difference in rates of decline between the two groups did not reach significance (*p* = 0.081, Supplementary Figure S3B). In the dementia subgroup, there were no significant associations between baseline ET and cognitive decline (Supplementary Figure S3 and Table S3).

### 3.4. Causal Mediation Analyses of CeVD and Neurodegeneration on the Associations between ET and Cognitive Decline

**Figure 3:**
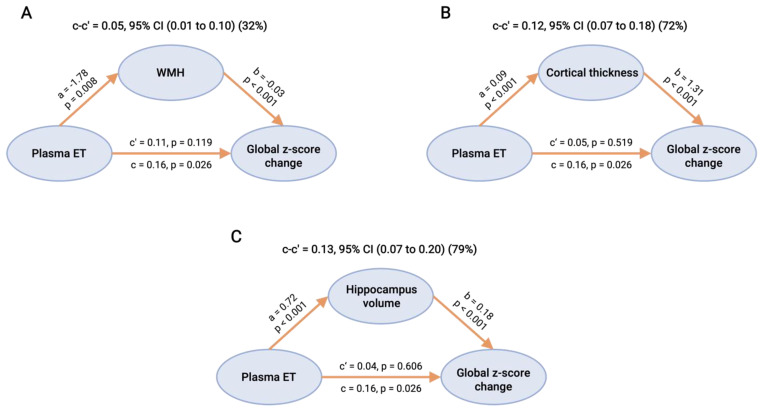
Mediation effects of brain MRI markers in the associations of baseline plasma ET levels with global cognitive decline. Causal mediation analyses of the total mediating effects (95% CI) of WMH (**A**), global cortical thickness (**B**), and hippocampus volume (**C**) are reported when there was a statistically significant mediation, as estimated by the difference between the total (path c) and direct (path c’) effect of plasma ET on cognitive change (c-c′). The direct effect of plasma ET on cognition (c’) was estimated by adjusting the association between plasma ET and cognitive change (total effect, c) for the mediator. The direct effect of plasma ET on the imaging markers is designated path a, while the direct effect of these markers on cognition is path b. All paths were adjusted by age, gender, education, APOE ε4 status, hypertension, diabetes, cardiovascular disease, and total intracranial volume. Abbreviations: CI = confidence interval, ET = ergothioneine, WMH = white matter hyperintensities.

We next examined whether the associations between baseline plasma ET and cognitive decline were potentially mediated by baseline CeVD and/or neurodegeneration markers as measured on MRI. Mediation analyses revealed that three MRI markers, namely, WMH, cortical thickness, and hippocampal volume, individually showed full mediation effects between baseline ET and global cognitive decline (32%, 72%, and 79% mediation, respectively), in that direct effects of ET on global cognition were no longer statistically significant after each marker was entered into the mediation model (Figure 3). In contrast, cortical infarcts and lacunes did not show significant mediation effects.

## 4. Discussion

Using a well-characterized, longitudinally assessed memory clinic cohort, we showed that lower ET levels were associated with (1) worse cognitive performance at baseline; (2) accelerated decline in global cognitive function as well as in multiple cognitive domains including memory, language, attention, executive function, and visuomotor speed over the 5-year follow-up, independent of baseline cognition; and (3) faster functional deterioration as indicated by an increase in CDR-SOB scores. When stratified by cognitive status, these associations were only evident in non-demented subjects (NCI + CIND groups). In addition, the associations between plasma ET and subsequent cognitive decline were largely explainable by neuroimaging measures of WMH and neurodegeneration. This suggests close links between decreasing ET levels and disease processes associated with cognitive impairment and dementia and also indicates that plasma ET could be a useful clinical biomarker for early detection of cognitive impairment and dementia.

ET has recently attracted attention due to its potential beneficial effects on cognition. Oral supplementation with ET was shown to enhance learning and memory performance in normal and in cognitively impaired mice models [23,25,26,44]. In humans, several cross-sectional observational studies have revealed a significant decline in ET levels in patients with MCI, AD, VaD, and other neurocognitive disorders [11,27,28,45,46]. Corroborating the observations from animal studies, several cohort studies have shown improvements in cognitive functions and reduced risk of developing cognitive impairments with dietary intake of mushrooms, known as one of the richest sources of ET [13,47,48,49,50,51]. Only one clinical study based in France has reported evidence of a link between peripheral ET and longitudinally assessed global cognitive decline [31]; however, this is the first study to comprehensively analyze the association between peripheral ET levels and individual cognitive domains as well as with known risk factors of cognitive decline (e.g., CeVD and brain atrophy). Moreover, considering that the intake of micronutrients and bioactive compounds is likely to be influenced by geographical and ethnic factors, investigations of the potential associations between ET and cognitive decline should be carried out in various populations and regions. Here, we investigated associations between plasma ET and longitudinal changes in a comprehensive range of both global and domain-specific cognitive functions, together with neuroimaging, in an Asian cohort. Our results provide additional evidence of a link between low ET levels and cognitive decline by showing that reduced ET levels are associated with impairments in multiple cognitive domains at baseline, as well as with a decline in global cognitive function for up to five years of follow-up. Furthermore, we observed that low ET levels were also associated with faster CDR-SOB increase, which may predict greater impairment in carrying out activities of daily living and poorer long-term clinical outcome.

Given that blood ET levels are known to decline with age and are also associated with other well-known risk factors for cognitive decline [11,27], we constructed multivariate models with adjustments for age, sex, education, APOE ε4 allele, and multiple vascular risk factors to account for potential confounding effects. The persistence of low plasma ET association with cognition even after covariate adjustments suggests that low plasma ET may be an independent risk factor for cognitive decline. Subgroup analyses revealed that associations between ET and cognition were exclusively found in the non-demented group, which has two major implications. Firstly, the potential beneficial effects of ET on cognition may be more pronounced in the early stages of disease, with implications on ET’s potential utility as an early diagnostic biomarker and as a potential therapeutic intervention. Secondly, the loss of significant associations within the dementia subgroup suggests a floor effect, given that patients clinically diagnosed with dementia are already in an advanced stage of disease, with a high burden of AD/CeVD pathology and prolonged, severe impairments of cognitive function [52,53].

The pathophysiological mechanisms underlying the effects of ET deficiency on cognitive decline are not fully understood. However, given ET’s established antioxidant and other cytoprotective properties, as well as its presence in the brain [14,54,55], it is likely that ET deficiency may lead to decreased brain antioxidant capacity with concomitant increases in oxidative stress. ET has been reported to exert neuroprotective effects in multiple models of neurodegeneration, possibly through the inhibition of neuroinflammation and oxidative stress, through chelation of metal cations (preventing promotion of redox activities), or by the preservation of mitochondrial function [23,25,26]. It has also been found that ET treatment can ameliorate β-amyloid-induced neurotoxicity and reduce toxic accumulation of amyloid in *Caenorhabditis elegans* and mice models [24,25,26]. Additionally, ET has been suggested to play important roles in vascular pathologies and has been associated with lower risk of cardiovascular disease, stroke, and mortality [56]. Endothelial dysfunction is known to be an early event in CeVD with links to oxidative stress [57]. ET has been shown to be taken up by endothelial cells where it exerts protective effects against oxidative damage, inhibits proinflammatory cytokine release, and attenuates monocyte recruitment [58,59,60]. Furthermore, we previously found that decreased plasma ET is associated with higher WMH and atrophy burden [11]. Given that both white matter lesions and neurodegeneration are strongly associated with subsequent cognitive decline [61,62,63], we tested the mediating effects of WMH and brain atrophy in relating ET to cognitive decline and showed through causal mediation analyses that associations between baseline ET levels and cognitive decline were predominantly mediated by WMH load as well as by the severity of cortical atrophy (Figure 3). We thus postulate that ET deficits are associated with cognitive decline via pathophysiological links with both CeVD as well as neurodegeneration.

Our hypothesis is further supported by the finding that associations between ET deficits and cognitive decline involve multiple cognitive domains, including both cortical function domains, i.e., memory, language, and executive function (a predominant feature of AD), as well as subcortical domains that are sensitive to the disruption of frontal-subcortical circuits, i.e., attention and visuomotor speed (as commonly observed in vascular-related cognitive impairment). Taken together, it is possible that ET deficiency is related to a loss of antioxidant protection, leading to a redox imbalance and increased susceptibility to oxidative damage associated with neurodegenerative and cerebrovascular disease processes, which could contribute to subsequent risk of cognitive and functional decline.

Several limitations of our study should be addressed in future studies. First, although we controlled for the major known or potential confounders, residual confounding remains a possibility due to a lack of comprehensive information on other factors that may affect baseline ET concentrations, such as possible genetic variations (e.g., polymorphisms in the gene encoding OCTN1) and dietary behaviors. Furthermore, it remains unclear whether the observed low ET levels are a pathogenic factor leading to inadequate oxidative defence or a consequence of antioxidant depletion due to disease-associated oxidative stress. If this is due to the former, then it is also not clear whether this decline in ET is due to a decline in transporter efficiency or expression, change in diet, or other possible mechanisms [64]. In this regard, it may be worthwhile conducting randomized controlled trials investigating the effectiveness of ET supplementation in the prevention of cognitive decline or dementia. Given that lower plasma ET levels are observed in APOE ε4 carriers, it would also be of interest to investigate whether ET has a genotype-specific effect on cognition, as previous studies have shown that the beneficial effect of antioxidant supplementation on cognition appeared to vary in polymorphic variants of APOE [65]. Additionally, our analyses were based on single determinations of baseline ET concentrations and did not account for longitudinal changes of ET within- and between-subjects. A follow-up study with ET measurements at multiple time-points may provide better temporal correlations between biomarker changes and clinical progression. Moreover, our results from the mediation analyses included only baseline plasma biomarker and neuroimaging data and thus should also be interpreted with caution because the assumed direction of causal relationship between the variables could be reversed or bidirectional. Lastly, whilst the focus of this study was on associations between ET and cognitive domains, it is of interest to study the association of ET levels with other longitudinally assessed blood or neuroimaging biomarkers for processes known to be involved in dementia pathophysiology, including amyloid aggregation, neuroinflammation, and oxidative stress. Future studies should also explore the role of ET in the cerebrospinal fluid in relation to other pathological biomarkers to further elucidate the underlying pathological mechanisms.

## 5. Conclusions

Following previous reports that low ET levels are associated with MCI and dementia, we now show, using a longitudinally assessed cohort of memory clinic subjects, that lower baseline ET levels are linked to higher rates of decline in multiple cognitive domains as well as increased severity of disease over a period of up to five years. The longitudinal cognitive consequences of low ET likely stem from its associations with cerebrovascular and neurodegenerative disease markers. Our findings suggest ET’s potential utility as a predictive biomarker of cognitive decline as well as a therapeutic supplement, especially for ET-deficient subjects in the predementia phases of disease. Further longitudinal studies and randomized trials of ET dietary supplementation may therefore be worthwhile.

## Figures and Tables

**Table 1:** Baseline demographics and disease factors of study participants.

Baseline Characteristics	Total Sample (*n* = 470)	Clinical Status		
		No Dementia (*n* = 281)	Dementia (*n* = 189)	*p* Value
Age, y, mean (SD)	73 (8)	72 (8)	75 (7)	<0.001 ***
Education, y, median (IQR)	6 (7)	9 (6)	5 (8)	<0.001 ***
Female, *n* (%)	248 (52.8)	141 (50.2)	107 (56.6)	0.171
APOE ε4 status (ε4+), *n* (%)	137 (29.1)	71 (25.3)	66 (34.9)	0.024 *
Body mass index, kg/m^2^, mean (SD)	23.8 (3.9)	24.0 (3.7)	23.4 (4.0)	0.112
Hypertension, *n* (%)	327 (70)	183 (65.4)	144 (77)	0.007 **
Hyperlipidemia, *n* (%)	348 (74.2)	210 (75.0)	138 (73)	0.630
Diabetes, *n* (%)	169 (36)	92 (32.7)	77 (40.7)	0.076
Cardiovascular disease, *n* (%)	57 (12.2)	32 (11.4)	25 (13.4)	0.530
MMSE score, median (IQR)	23 (10)	26 (5)	16 (7)	<0.001 ***
CDR-SOB score, median (IQR)	1.0 (5.0)	0 (1.0)	6.0 (5.0)	<0.001 ***
Cerebrovascular disease markers ^¶^				
Cortical infarcts, *n* (%)	53 (12.3)	25 (9.3)	28 (17.4)	0.013 *
Lacunes ≥ 2, *n* (%)	58 (13.5)	28 (10.4)	30 (18.6)	0.016 *
WMH, ARWMC ≥ 8, *n* (%)	175 (40.7)	82 (30.5)	93 (57.8)	<0.001 ***
Neurodegenerative markers				
Global cortical thickness, mm, mean (SD)	2.3 (0.1)	2.3 (0.1)	2.2 (0.1)	<0.001 ***
Hippocampus volume, ml, mean (SD)	6.4 (1.2)	6.8 (1.0)	5.6 (1.1)	<0.001 ***
Total intracranial volume, ml, mean (SD)	1095 (112)	1099 (108)	1088 (121)	0.335
Ergothioneine, nM, median (IQR)	784 (855)	896 (882)	588 (549)	<0.001 ***

MRI measures of CeVD and neurodegeneration were available for 430 participants. ^¶^ Cut-offs of counts/scores for definitions of significant CeVD have been previously described [43]. *, **, and *** indicate statistically significant at *p* < 0.05, *p* < 0.01, and *p* < 0.001, respectively, based on Student t-test (age, BMI, neurodegenerative markers); Chi-Square (gender, APOE ε4 status, hypertension, diabetes, cardiovascular diseases, hyperlipidemia, CeVD markers); and Mann–Whitney U test (education, MMSE, CDR-SOB, ergothioneine). Abbreviations: CDR-SOB = Clinical Dementia Rating Sum-of-Boxes, CIND = cognitive impairment, no dementia, NCI = no cognitive impairment, BMI = body mass index, ml = milliliters, mm = millimeters, *n* = number of cases, SD = standard deviation, IQR = interquartile range, CeVD = cerebrovascular disease, ARWMC = age-related white matter changes, MMSE = Mini-Mental State Examination.

**Table 2:** Baseline associations between plasma ET and cognitive performance.

	Mean Difference in Baseline Cognitive Z-Scores per Unit Increase in Log-Transformed Plasma ET Levels	
	β (95% CI) *	*p* Value
Global	2.02 (1.36, 2.68)	<0.001
Executive function	1.44 (0.87, 2.01)	<0.001
Attention	0.56 (0.21, 0.91)	0.002
Language	2.90 (1.58, 4.22)	<0.001
Visuospatial function	1.54 (0.99, 2.10)	<0.001
Visuomotor speed	0.79 (0.48, 1.09)	<0.001
Memory	1.32 (0.91, 1.73)	<0.001

* Linear regression models with mean differences (β) and 95% CI were used for log-transformed plasma ET levels as a continuous independent variable. All values were adjusted for age, gender, education, APOE ε4 status, hypertension, diabetes, and cardiovascular disease. Interpretation: significant β (*p* < 0.05) indicates an increase in cognitive z-scores based on the β value for every 10-fold (1 unit of log 10 [plasma ET]) increase in plasma ET levels at baseline. Abbreviations: CI = confidence interval, ET = ergothioneine.

**Table 3:** Effects of baseline plasma ET on CDR-SOB progression and global/domain-specific cognitive decline.

	Mean Difference in the Rate of Change of Outcome Scores per Unit Increase in Log-Transformed Plasma ET Levels	
	β (95% CI) *	*p* Value
CDR-SOB ^a^	−0.55 (−0.87, −0.22)	0.001
Neuropsychological z-scores ^b^		
Global	0.23 (0.06, 0.40)	0.008
Executive function	0.12 (0.02, 0.23)	0.019
Attention	0.12 (0.04, 0.21)	0.004
Language	0.49 (0.03, 0.94)	0.037
Visuospatial function	0.07 (−0.04, 0.18)	0.210
Visuomotor speed	0.05 (0.01, 0.09)	0.028
Memory	0.10 (0.03, 0.17)	0.007

* Mean differences (β coefficients) of the interaction term ET × time and 95% CI were derived from linear mixed-effect models with log-transformed plasma ET levels included as a continuous variable. All values adjusted for age, gender, education, APOE ε4 status, hypertension, diabetes, cardiovascular disease, and baseline MMSE. Interpretation: ^a^ significant β (*p* < 0.05) < 0 indicates a decrease in the rate of progression in CDR-SOB scores based on the β value for every 10-fold (1 unit of log 10 [plasma ET]) increase in plasma ET levels at baseline. ^b^ significant β (*p* < 0.05) > 0 indicates a decrease in the rate of decline in cognitive z-scores based on the β value for every 10-fold (1 unit of log 10 [plasma ET]) increase in plasma ET levels at baseline. Abbreviations: CDR-SOB = Clinical Dementia Rating Sum-of-Boxes, CI = confidence interval; ET = ergothioneine, MMSE = Mini-Mental State Examination.

## Data Availability

The data presented in this study are available in the article and supplementary material.

## Supplementary Materials

The following supporting information can be downloaded at: https://www.mdpi.com/article/10.3390/antiox11091717/s1, Table S1: Summary of the neuropsychological battery and component tests; Table S2: Associations between plasma ET and baseline cognitive performance in a memory clinic cohort; Table S3: Associations of plasma ET with longitudinal cognitive and functional decline in a memory clinic cohort; Figure S1: Selection of study participants; Figure S2: Baseline measures of cognitive z-scores for high and low ET subjects stratified by clinical subgroups; Figure S3: Functional and cognitive trajectories in relation to baseline plasma ET stratified by clinical subgroups; Figure S4: Associations of baseline plasma ET with functional outcomes (≥ 1-point increase in CDR-SOB scores).
