# Supplementary material for: Low Plasma Ergothioneine Predicts Cognitive and Functional Decline in an Elderly Cohort Attending Memory Clinics
Source: Antioxidants (Basel). 2022 Aug 30;11(9):1717. doi: 10.3390/antiox11091717 (PMC9495818; doi:10.3390/antiox11091717)
Supplement: Supplementary file 1 [file antioxidants-11-01717-s001.zip › antioxidants-1828689-supplementary.pdf]

Wu et al. Supplementary information

Supplementary Table S1: Summary of Neuropsychological Battery and Component Tests

Cognitive Domain	Component Test(s)
1. Executive Function:	Verbal fluency [1], Color trails test A&B [2]
2. Attention:	Digit span forward and backward [3]
3. Language:	15-item modified Boston Naming Test [4]
4. Visuomotor Speed:	Symbol Digit Modalities Test [5]
5. Visuospatial Function:	Rey Complex Figure Test-copy [6]
6. Memory:	Rey Complex Figure Test-immediate/delayed recall and recognition [6], Hopkins Verbal Learning Test-immediate/delayed test recall and recognition [7]

References

1. McKhann, G., et al., *Clinical diagnosis of Alzheimer's disease: report of the NINCDS-ADRDA Work Group under the auspices of Department of Health and Human Services Task Force on Alzheimer's Disease.* Neurology, 1984. **34**(7): p. 939-44.
2. D'Elia, L., et al., *Color Trails Test. Odessa, FL: Psychological Assessment Resources.* Inc Google Scholar, 1996.
3. Wechsler, D., *Subtest Administration and Scoring. WAIS–IV: Administration and Scoring Manual.* San Antonio, TX: The Psychological Corporation, 2009: p. 87-93.
4. Mack, W.J., et al., *Boston Naming Test: shortened versions for use in Alzheimer's disease.* Journal of gerontology, 1992. **47**(3): p. P154-P158.
5. Smith, A., *Symbol digit modalities test.* 1973: Western psychological services Los Angeles.
6. Meyers, J., *Meyers scoring system for the Rey complex figure test and the recognition trial.* Psychological Assessment Resources, Odessa, FL, 1994.
7. Brandt, J., *The Hopkins Verbal Learning Test: Development of a new memory test with six equivalent forms.* The clinical neuropsychologist, 1991. **5**(2): p. 125-142.

Supplementary Table S2: Associations between plasma ET and baseline cognitive performance in a memory clinic cohort

	Mean difference in baseline cognitive z-scores per unit increase in log-transformed plasma ET levels			
	Non-demented (N=281)		Dementia (N=189)	
	β (95% CI)*	P value	β (95% CI)*	P value
Global	**0.90 (0.38, 1.42)**	**<0.001**	1.00 (-0.05, 2.05)	0.061
Executive function	**0.64 (0.04, 1.23)**	**0.038**	0.51 (-0.24, 1.25)	0.184
Attention	0.42 (-0.01, 0.86)	0.058	0.26 (-0.34, 0.86)	0.392
Language	0.73 (-0.03, 1.49)	0.061	1.93 (-0.81, 4.67)	0.167
Visuospatial function	**0.64 (0.07, 1.22)**	**0.029**	**1.01 (0.13, 1.89)**	**0.025**
Visuomotor speed	**0.38 (0.02, 0.74)**	**0.037**	0.25 (-0.08, 0.58)	0.136
Memory	**1.01 (0.54, 1.47)**	**0.001**	0.24 (-0.15, 0.64)	0.223

*Linear regression models with mean differences (β) and 95% CI were used for log-transformed plasma ET levels as a continuous independent variable. All values adjusted for age, gender, education, APOE ε4 status, hypertension, diabetes, and cardiovascular disease. ET: ergothioneine.

Interpretation: Significant β **(bold text)** indicates an increase in cognitive z-scores by β value for every 10-fold (1 unit of log 10 [plasma ET]) increase in plasma ET levels at baseline.

Supplementary Table S3: Associations of plasma ET with longitudinal cognitive and functional decline in a memory clinic cohort

	Mean difference in rate of change of outcome scores per unit increase in log-transformed plasma ET levels			
	**Non-demented (N=281)**		**Dementia (N=189)**	
	**β (95% CI)***	**P value**	**β (95% CI)***	**P value**
CDR-SOB scores	-0.32 (-0.67, 0.03)	0.072	0.15 (-0.45, 0.75)	0.617
Neuropsychological z-scores				
Global	0.13 (-0.02, 0.27)	0.086	-0.23 (-0.59, 0.14)	0.218
Executive function	0.07 (-0.04, 0.18)	0.226	-0.11 (-0.31, 0.09)	0.263
Attention	0.05 (-0.03, 0.13)	0.254	0.04 (-0.16, 0.23)	0.690
Language	0.21 (-0.11, 0.52)	0.206	-0.72 (-1.77, 0.33)	0.175
Visuospatial function	0.06 (-0.06, 0.18)	0.347	-0.20 (-0.42, 0.02)	0.070
Visuomotor speed	0.05 (0.00, 0.10)	0.056	-0.02 (-0.11, 0.06)	0.552
Memory	0.04 (-0.05, 0.13)	0.407	0.05 (-0.05, 0.14)	0.367

* Mean differences (β coefficients) of the interaction term ET × time and 95% CI were derived from linear mixed-effect models with log-transformed plasma ET levels included as a continuous variable. All values adjusted for age, gender, education, APOE ε4 status, hypertension, diabetes, cardiovascular disease, and baseline MMSE. CDR-SOB: Clinical Dementia Rating Sum-of-Boxes; CI: confidence interval; ET: ergothioneine

Supplementary Figure S1: Selection of study participants

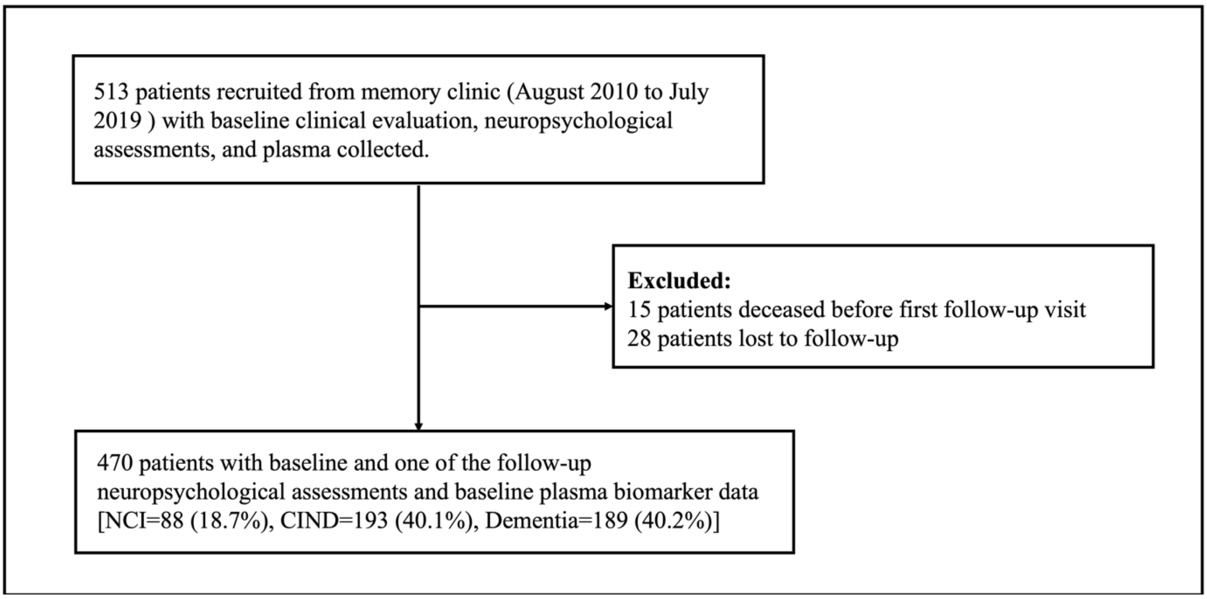

CIND, cognitive impairment no dementia; NCI, no cognitive impairment.

Supplementary Figure S2: Baseline measures of cognitive z-scores for high and low ET subjects stratified by clinical subgroups

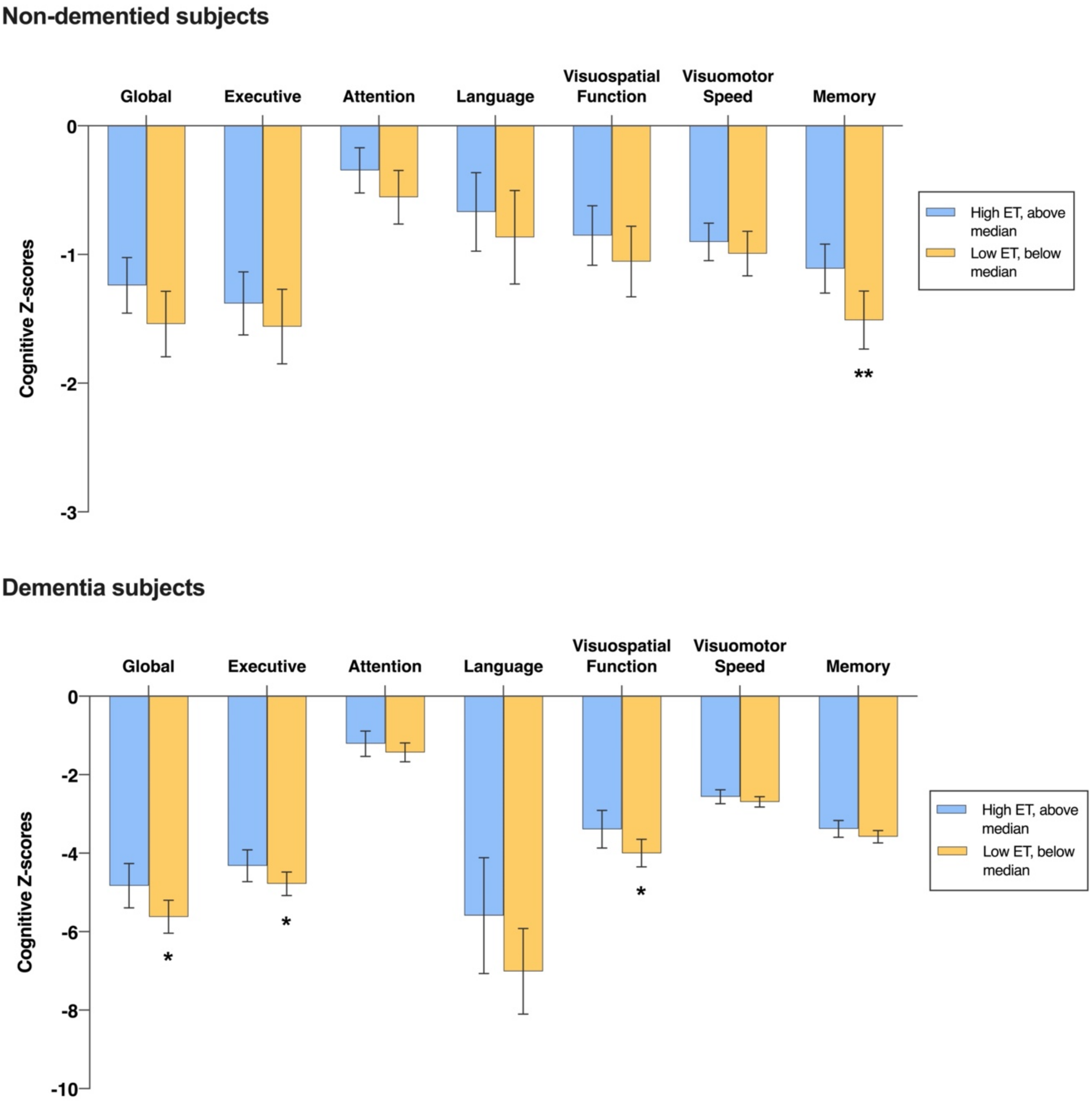

Baseline measures of global and domain-specific cognitive z-scores for high ET and low ET patients (using group median as a cut-off point) are presented separately for non-demented group (A) and dementia group (B). Bar graphs represent the estimated marginal means from the linear regression models, with error bars representing 95% confidence interval. Analyses are shown with adjustments for age, gender, education, APOE ε4 status, hypertension, diabetes, and cardiovascular disease. ET: ergothioneine. *, and ** indicate significant association by multiple linear regression at $p<0.05$ and $p<0.01$, respectively.

Supplementary Figure S3: Functional and cognitive trajectories in relation to baseline plasma ET stratified by clinical subgroups

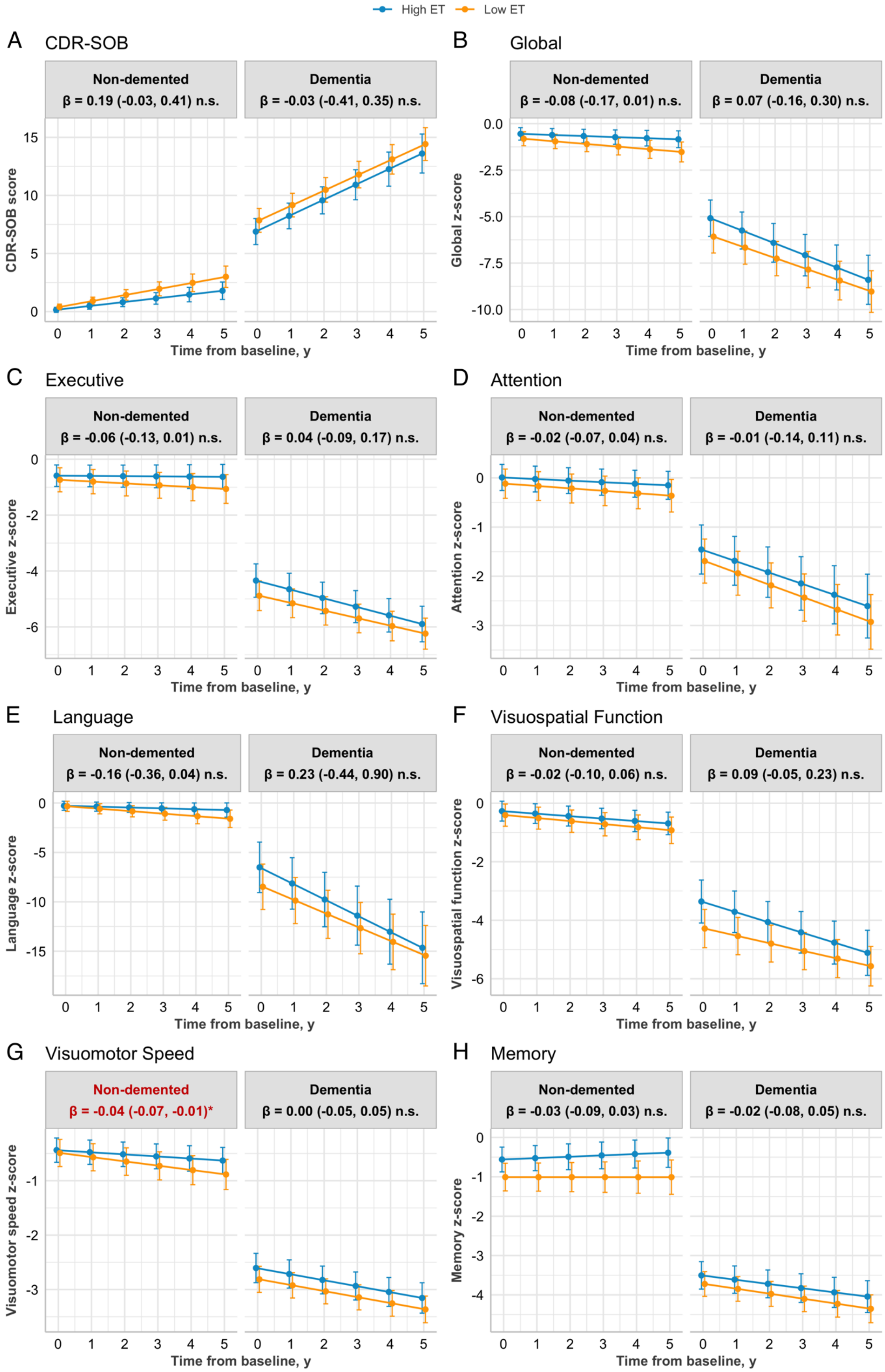
High ET
Low ET
A CDR-SOB
Non-demented β = 0.19 (-0.03, 0.41) n.s.
Dementia β = -0.03 (-0.41, 0.35) n.s.
CDR-SOB score
B Global
Non-demented β = -0.08 (-0.17, 0.01) n.s.
Dementia β = 0.07 (-0.16, 0.30) n.s.
Global z-score
C Executive
Non-demented β = -0.06 (-0.13, 0.01) n.s.
Dementia β = 0.04 (-0.09, 0.17) n.s.
Executive z-score
D Attention
Non-demented β = -0.02 (-0.07, 0.04) n.s.
Dementia β = -0.01 (-0.14, 0.11) n.s.
Attention z-score
E Language
Non-demented β = -0.16 (-0.36, 0.04) n.s.
Dementia β = 0.23 (-0.44, 0.90) n.s.
Language z-score
F Visuospatial Function
Non-demented β = -0.02 (-0.10, 0.06) n.s.
Dementia β = 0.09 (-0.05, 0.23) n.s.
Visuospatial function z-score
G Visuomotor Speed
Non-demented β = -0.04 (-0.07, -0.01)*
Dementia β = 0.00 (-0.05, 0.05) n.s.
Visuomotor speed z-score
H Memory
Non-demented β = -0.03 (-0.09, 0.03) n.s.
Dementia β = -0.02 (-0.08, 0.05) n.s.
Memory z-score
Time from baseline, y

Trajectories of CDR-SOB scores (A) and global/domain-specific cognitive z-scores (B-H) in relation to baseline plasma ET status are presented separately for Non-demented group and Dementia group. Estimated means of cognitive scores with 95% CI are presented for those with high baseline ET (above group median; blue lines) and low baseline ET (below group median; orange lines). β-coefficients (95% CI) were derived from linear mixed-effect models adjusted for age, gender, education, APOE ε4 status, hypertension, diabetes, cardiovascular disease, and baseline MMSE. CDR-SOB: Clinical Dementia Rating Sum-of-Boxes; ET: ergothioneine; n.s.: not significant. * (in red text) indicates significantly different rate of change in cognitive scores between low ET vs high ET patients at $p<0.05$.

Supplementary Figure S4: Associations of baseline plasma ET with functional outcomes (≥1-point increase in CDR-SOB scores)

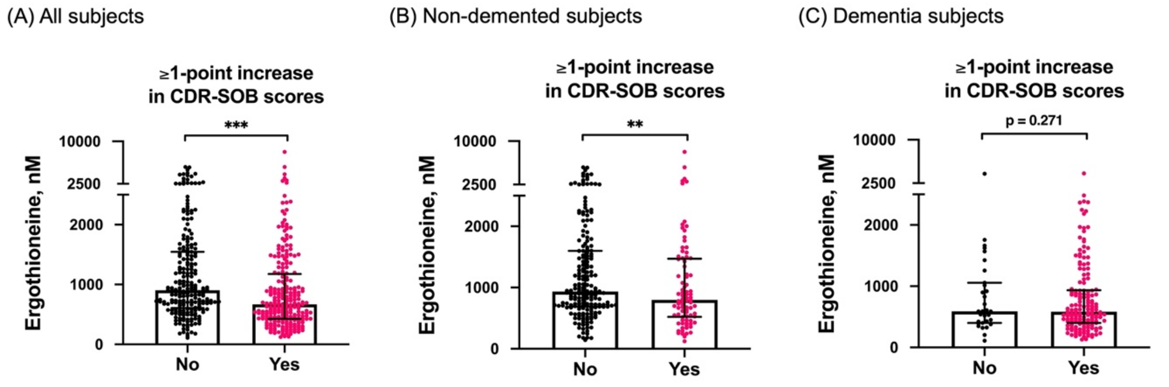

(A) All subjects

	OR (95% CI)	P value
Model 1	0.11 (0.05, 0.23)	<0.001
Model 2	0.16 (0.07, 0.39)	<0.001

(B) Non-demented subjects

	OR (95% CI)	P value
Model 1	0.20 (0.07, 0.54)	0.001
Model 2	0.21 (0.06, 0.76)	0.018

(C) Dementia subjects

	OR (95% CI)	P value
Model 1	0.38 (0.05, 2.75)	0.338
Model 2	0.41 (0.05, 3.44)	0.413

Associations of baseline plasma ET levels with 1-point or more increase in CDR-SOB scores during follow-up in all subjects (A), non-demented subgroup (B) and dementia subgroup (C). Bar graphs show medians and interquartile ranges (IQR). Mann-Whitney U tests were performed, with significant p values indicated with asterisks: **p < 0.01, ***p < 0.001. Reported odds ratios (ORs) and 95% CI were derived from binary logistic regression models with log-transformed plasma ET levels included as a continuous independent variable. Model 1: unadjusted. Model 2: adjusted for age, gender, education, APOE ε4 status, hypertension, diabetes, and cardiovascular disease. CDR-SOB: Clinical Dementia Rating Sum-of-Boxes, ET: ergothioneine.

Interpretation: Significant OR < 1 (p<0.05) indicates that each 10-fold (1 unit of log 10 [plasma ET]) increase in plasma ET level was associated with (1-OR)*100% lower chance of getting the respective cognitive outcome.
